# ‘If I Don’t Have My Support Worker in the Room…’: A Multi-perspective Mixed Methods Study of Remote Daily Living Support for Neurodivergent Young Adults

**DOI:** 10.1007/s10803-024-06425-z

**Published:** 2024-06-05

**Authors:** Maria Löthberg, Eda Wirström, Jenny Meyer, Sonya Girdler, Sven Bölte, Ulf Jonsson

**Affiliations:** 1 Center of Neurodevelopmental Disorders (KIND), Centre for Psychiatry Research, Department of Women’s and Children’s Health, Karolinska Institutet & Stockholm Health Care Services, Region Stockholm, Stockholm, Sweden; 2 Department of Social Psychiatry and Individual Support, Municipality of Uppsala, Uppsala, Sweden; 3 Child and Adolescent Psychiatry, Stockholm Health Care Services, Region Stockholm, Stockholm, Sweden; 4 Curtin Autism Research Group, Curtin School of Allied Health, Curtin University, Perth, Australia; 5 Department of Medical Sciences, Child and Adolescent Psychiatry, Uppsala University, Uppsala, Sweden

**Keywords:** ADHD, Autism, Daily living, Remote support, Social services

## Abstract

**Purpose:**

Information technology is increasingly being employed for providing support and interventions in disability and health service contexts. This study aimed to investigate service users’ and support workers’ perspectives on remote support in daily living for young adults with neurodevelopmental conditions.

**Methods:**

Using a convergent mixed methods approach, we integrated qualitative and quantitative findings from survey responses and focus groups. Young service users (aged 18 to 29) diagnosed with ADHD and/or autism (*n* = 35) and support workers (*n* = 64) from four municipalities in Sweden responded to a survey designed to tap into their lived experiences and views. The topic was explored further in focus groups with young service users (*n* = 7) and support workers (*n* = 3). Open-ended survey questions were analyzed using qualitative content analyses and complemented with information from the focus groups, while closed survey questions were summarized descriptively. Inferences were merged in a joint display.

**Results:**

While participants reported having access to digital devices, service routines for remote contact were not in place. Service users were more hesitant than support workers in endorsing remote support, expressing concerns that this approach would be inferior to in-person support (e.g., owing to miscommunications and insufficient social and emotional contact). Still, both groups expressed that remote contact may at times be a beneficial complement to in-person meetings, increasing accessibility and user choice.

**Conclusion:**

Service providers planning to implement remote support elements should explore the demand, acceptability, and organizational readiness for this approach. Moving forward, user engagement will be crucial to meet individual preferences, values, and needs.

**Supplementary Information:**

The online version contains supplementary material available at 10.1007/s10803-024-06425-z.

Transition to adulthood is a transformative period that is defined by escalating expectations regarding assuming adult roles and independence (Arnett, 2000; Arnett & Padilla-Walker, 2015). This period is commonly marked by the achievement of many life milestones including going to university, getting a job, leaving home, and building new relationships (e.g., Speder et al., 2013). Young neurodivergent adults with conditions such as autism and ADHD may find this period particularly challenging (Elster & Parsi, 2020; Roper, 2023). Research using the WHO framework International Classification of Functioning, Disability, and Health (ICF) (World Health Organization, 2001) suggests that this group experiences functional challenges in several domains (e.g., decision-making, problem-solving, daily routines, housework, distributing energy and drive on different objectives, and managing basic interpersonal interactions) (Bölte et al., 2018, 2019). Support may consequently be needed in multiple life areas, such as finances, housing, education, employment, transportation, and health care (e.g., Elster & Parsi, 2020).

National guidelines in Sweden have recently highlighted support in daily living as a high-priority service for neurodivergent adults (Swedish National Board of Health and Welfare, 2022b). The most frequently granted support service to young adults is a form of service called housing support, which is provided under the Swedish Social Services Act (Swedish National Board of Health and Welfare, 2023b). This service includes the provision of practical, educational, and social support to enable individuals to manage their daily lives in ordinary housing (Swedish National Board of Health and Welfare, 2010). A recent qualitative study (Löthberg et al., 2023) of support workers’ experience revealed that increasingly elements of this support are provided remotely, via digital devices. The support workers described that the use of digital technology had accelerated during the COVID-19 pandemic and that these changes in service provision had resulted in new insights into how support can be tailored to meet different needs. New ways of using and combining remote support with in-person meetings were seen as a valuable addition to the traditional model of service.

In the broader perspective, scientific advances have enabled the use of information technology in healthcare and social services (e.g., Milne-Ives et al., 2020). Digital platforms are commonly employed in remotely delivering advice, support, and care to service users, which was accelerated by the COVID-19 pandemic (Fang et al., 2022; World Health Organization, 2020). While the proposed benefits of digitally delivering healthcare interventions include improved accessibility (Kazlauskas et al., 2020; Sehlin et al., 2018; Venturo-Conerly et al., 2022) and cost-effectiveness (Andersson & Titov, 2014), concerns have been raised that these approaches are less client-centered (Stevens et al., 2019) and limit social contacts among users (Fang et al., 2022). WHO guidelines on digital health interventions suggest that health care services provided at a distance may improve outcomes, and that clients may feel that this improves their independence and self-care. On the other hand, qualitative evidence indicates that health workers have concerns, expressing that some clients still need face-to-face contact, noting that changes in the relationship and communication may lead to poorer quality care. In addition, challenges concerning the feasibility of digital interventions were highlighted, including the issue of maintaining data privacy and technical issues. Overall, the guidelines stress that delivery of health care services at a distance should complement and enhance, rather than replace, face-to-face services (World Health Organization, 2019).

Today, younger generations are frequently described as ‘digitally native’ (e.g., Dahl et al., 2018) and may therefore be more ready to accept, or even prefer digital health care and support services. The use of digital technology in providing support and interventions to neurodivergent young people may have significant potential (Khan et al., 2019), increasing accessibility and simplifying communication (Sehlin et al., 2018). Still, some concerns have been raised regarding their effectiveness in enabling young adults to learn and retain skills (Chen et al., 2022; Khan et al., 2019).

Despite the increasing use of remote elements in daily living support, to date no research has investigated how this shift is perceived by young service users and the support workers. The objective of this study was to investigate neurodivergent young service users’ (ages 18 to 29 years) and support workers’ perspectives on remote support. A survey was designed to tap into the lived experience and views of the respondents and was complemented by focus group discussions. By accessing insights into specific needs, possibilities, and barriers, we aimed to gain a better understanding of the experiences encountered in implementing remote elements of support in daily living.

## Methods

### Design

**Fig. 1:**
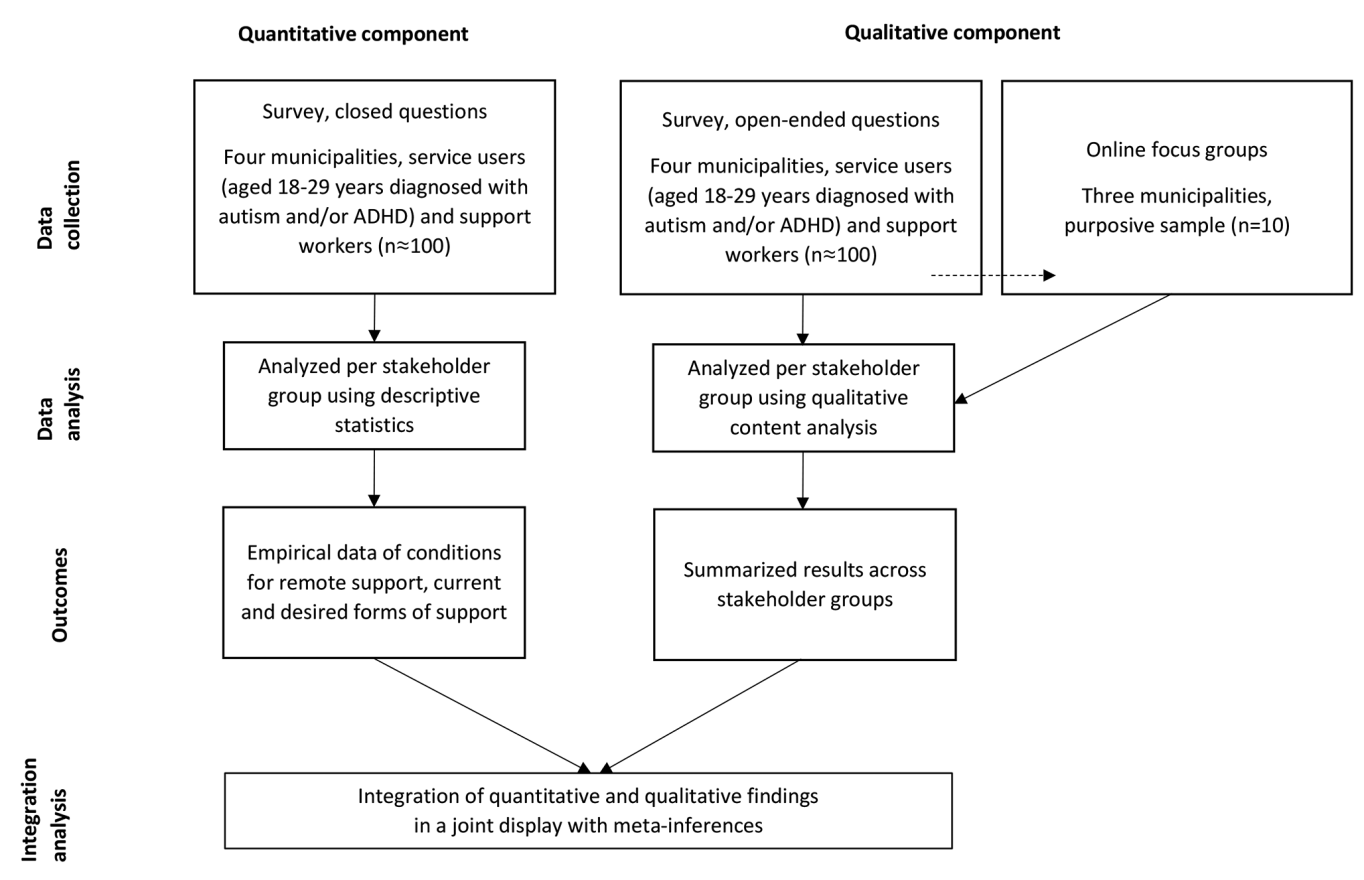
Visual depiction of the convergent mixed methods design

This study was the first step in a larger project aiming to increase young service user engagement in the support service. A convergent mixed methods design (Creswell, 2022) was employed with the goal of gaining knowledge about young service users and support workers’ views on, and readiness for, remote support (see Fig. 1). This design was chosen to allow for both quantitative and qualitative analyses, which were integrated to make inferences from the merged data (Creswell, 2022; Guetterman et al., 2021). Two data sources were used: (1) a survey including closed and open-ended questions; and (2) online focus group discussions. Multi-perspective data, in this case, data collected from two stakeholder groups (neurodivergent young service users and support workers), was collected to access multiple descriptions (Doyle et al., 2020; Åkerblad et al., 2021). Members of the research group with lived experience of support in daily living, from both a service user and support worker perspective, read and commented on the final manuscript. Neurodivergent individuals were not directly involved in the design of the study or the development of the survey. The study was performed in line with the principles of the Declaration of Helsinki and was approved by the Swedish Ethical Review Authority (2021-06924-01).

### Context

Housing support is a type of support in daily living, typically delivered by municipal social services in Sweden. The objective of this service is to improve the quality of life and promote equality for individuals with mental, behavioral, and neurodevelopmental conditions living in ordinary housing in Sweden (Swedish National Board of Health and Welfare, 2022a). Consequently, those living in special housing with services for people with functional impairment (e.g., supported housing) do not qualify for this specific service. The support service is organized and managed within each municipality. Designed as a needs-based service, social care administrators initially assess a potential client’s eligibility, based on their individual profile and needs. The decision is usually underpinned by an interview with the client and information obtained from clinical assessments and medical statements.

The support is delivered by public and/or private service providers. A support plan, containing relevant information about the service user and the scope and area of granted support, is forwarded by the care administrator to the service providers. The frequency of support varies according to the individual’s needs, from as little as once a month to several times a day. The service is typically carried out in the service user’s home, but can also be provided at other locations, depending on the service users’ preferences and needs. Support workers are predominantly women and commonly have an educational background in nursing and social care (e.g., assistant nurse), while some have higher education in behavioral science or social work (Statistics Sweden, 2022; Swedish National Board of Health and Welfare, 2010, 2022a).

Service users are heterogeneous in terms of mental, behavioral, and neurodevelopmental conditions, with anxiety disorder, depression, ADHD, schizophrenia, autism, and personality disorders being the most common conditions. A smaller proportion (approximately 5%) of users have an intellectual disability (Swedish National Board of Health and Welfare, 2019). Nationally, approximately 30 000 individuals are granted this service, with users equally distributed between males and females, and about one third being young adults (Swedish National Board of Health and Welfare, 2023b). It has been reported that many who would qualify for this support service do not apply, limiting understanding as to the true size of the target population (Autism Sverige, 2023; Swedish National Board of Health and Welfare, 2023a).

### Participants

#### The Survey

Using purposive sampling, four municipalities were selected based on demography (one rural and three urban) and type of provider (one private and three public). Service users aged 18 to 29 years with a diagnosis of autism and/or ADHD and support workers who had worked with this target group in the last two years were eligible to participate in the survey. Support workers were asked to invite their clients diagnosed with autism or ADHD to participate. Thereby, only young adults with a known clinical diagnosis of autism and ADHD were invited. In the survey, the respondents themselves confirmed the diagnosis. Based on the municipalities’ estimates, the target population comprised approximately 200 service users and 200 support workers. A total of 35 young service users and 64 support workers completed the survey. Participant characteristics for the total sample and stratified by diagnosis are described in Table 1 and Online resource Table S1.

**Table 1:** Demographic characteristics and experience with housing support among service users and support workers responding to the survey

		Total	Experience with housing support		
*Service users* ^*a*^			*≈ 1 year*	*≈ 2–3 years*	*≈ 4–10 years*
Gender	All	35	12	17	6
	Women	21	7	11	3
	Men	10	2	5	3
	Non-binary	4	3	1	0
Age^b^	18–24 years	9	5	3	1
	25–29 years	25	7	13	5
Diagnosis^c^	Autism	16	8	5	3
	ADHD	10	3	7	0
	Autism and ADHD	8	1	5	2
*Support workers* ^*d*^			*1–5 years*	*6–10 years*	*11 ≥ 20 years*
Gender	All	64	24	16	23
	Women	50	18	12	19
	Men	14	6	4	4
Age^b^	25–35 years	14	10	3	0
	36–45 years	11	5	3	3
	46–55 years	20	6	3	11
	56–65 years	18	3	7	8

^*a*^ *11 web-based responses and 24 on paper*

^*b*^ *One participant did not state their age*

^*c*^ *One participant did not state their diagnosis*

^*d*^ *One participant did not state years of work experience*

#### The Focus Groups

Purposive sampling was also used to recruit participants for three online focus groups (autistic service users; service users with ADHD; and support workers) using the same eligibility criteria as for the survey with the additional requirement that participants should be prepared to take part in online group discussions. The aim was to create focus groups consisting of one participant from each municipality. Since one municipality declined to participate in the focus groups, participants were ultimately nominated by three municipalities. Nominated support workers (*n* = 3) were sent an information letter and returned an informed consent form. Support workers were instructed to provide eligible service users with oral and written information and assist them in returning their informed consent forms as necessary. Participating service users (*n* = 7) were allocated into two groups based on their primary condition, with some participants having co-occurring autism and ADHD. The two groups included three participants each, with one additional participant with co-occurring autism and ADHD ultimately deciding to contribute through telephone calls. Participating young service users were living in ordinary housing, were aged 21 to 29 years, and had between 1 and 8 years of experience in receiving support in their daily living. A range of educational levels (i.e., primary school, secondary school, university, and vocational training) and current occupations (i.e., part-time, sick leave, student, volunteer, and self-employed) were represented. To ensure the participants anonymity, more detailed individual-level information could not be disclosed. The group of support workers consisted of two women and one man aged 34 to 60 years, with 9 to 25 years of work experience in the support services. They all had a higher education degree (i.e., social work, psychology, and behavioral science).

### Materials

#### The Survey

An anonymous survey was constructed using the survey tool Artologik Survey & Report (version 5). Eight closed questions mapped participants’ characteristics, access to digital devices, access to support in the use of digital devices, and use of digital devices in their support. Ten questions focusing on areas for support were constructed using branching logic (where additional questions were based on the respondent’s answers) to capture current formats of support and desired future support formats. These ten questions ended with the option to elaborate on their responses in free text. To allow participants to provide additional information on remote support as a facilitator or barrier to support in daily living, the survey concluded with two open-ended free text questions. An example from the survey is shown in the Online Resource Table S2.

In line with the WHO definition of telehealth (World Health Organization, 2023), remote support was defined as support provided from a remote location using technological devices, such as smartphones or computers, without the direct involvement of an on-site support worker. Ten support domains, common for both autism and ADHD, were selected for survey questions using the brief ICF Core Sets for autism (Bölte et al., 2019) and ADHD (Bölte et al., 2018). The selected support domains have previously been pinpointed as salient areas of functioning for these groups (Bölte et al., 2018, 2019), and harmonize with the support needs of young adults with support in daily living in general (Andersson & Gustafsson, 2014; Brolin, 2016; Löthberg et al., 2023). Survey questions were operationalized for the following ICF codes: d175 + d177 (Solving problems + Making decisions); d230 (Carry out daily routine); d240 (Handling stress and other psychological demands); d570 (Looking after one’s health); d640 (Doing housework); d710 (Basic interpersonal interactions); d870 (Economic self-sufficiency); d920 (Recreation and leisure); b130 (Energy and drive functions); and e330 (People in positions of authority).

### Procedure

#### The Survey

Managers in the four participating municipalities distributed an information letter addressed to the support workers describing the study and procedures, including a web link and a QR code to the web survey. Eligible young service users were informed orally by their support worker and were given a corresponding information letter. The young service users could respond to the survey online or on paper. Web surveys were kept open between September 16 and November 16, 2022. Surveys on paper were accepted until December 1, 2022.

#### The Focus Groups

Focus groups were scheduled to meet on two occasions lasting about three hours each, including breaks. The password-protected meetings were held online via Zoom (Zoom Video Communications, Inc.). The meetings were led by ML and EW, who have several years of work experience in providing support in daily living for neurodivergent young adults. Extensive field notes were taken, and pictures were taken of notes made on a digital whiteboard during meetings.

The meetings followed a fixed agenda. The first meeting focused on sharing experiences and views on the use of different forms of support, and the second meeting focused on how remote support may be used within support domains selected from the ICF core sets. Ahead of the second meeting, the list of support domains was sent to all participants. One service user contributed through two 1 to 1.5-hour telephone calls with the first author (ML) following the same agenda. The responses from the survey were used in preparing for the focus groups, enabling focus group leaders to raise relevant issues that did not emerge spontaneously. Focus groups were held from February to April 2023. All participants received financial compensation for their time.

### Analysis

#### Quantitative

Responses to the closed survey questions were summarized separately for young service users, the total sample and categorized by diagnostic group, and for support workers using descriptive statistics.

#### Qualitative

The free-text responses from the survey were analyzed separately for each stakeholder group, using inductive qualitative content analysis (Graneheim et al., 2017; Graneheim & Lundman, 2004). Meaning units consisting of a few words, a sentence, or a whole statement, were identified and labeled with a code while retaining the manifest content of each unit. To capture similarities and differences, the categorization process began with two authors (ML and EW) collaboratively grouping the codes into subcategories. The subcategories were then used as a preliminary framework to summarize the notes from the focus group meetings per stakeholder group, thus enriching and expanding the subcategories. These summaries were then read and discussed by four authors (ML, EW, JM, and UJ). Next, the two stakeholder perspectives were brought together and grouped for each subcategory by combining the data from the survey and the focus groups. Through discussions in the team (ML, EW, JM, and UJ), the original subcategories were then revised in an iterative process (Elo et al., 2014; Graneheim et al., 2017). The final results were reviewed to detect possible differences between diagnostic groups (i.e., autism, ADHD, or both).

#### Integration

The qualitative and quantitative results were merged to compare, examine, and relate the results and thereby enable meta-inferences (Creswell, 2022; Guetterman et al., 2021). First, two of the authors (ML and UJ) discussed major inferences from the qualitative and quantitative analyses. Qualitative and quantitative findings were listed side by side in a joint display and matched with themes drawn from the World Health Organization’s framework for quality elements in health services (World Health Organization, 2018). The joint display analysis was an iterative process conducted by the two authors, transforming the findings into key points on a similar level of aggregation and generating meta-inferences. Themes from the framework were selected and modified as necessary, to represent our findings. The joint display was subsequently refined after discussions within the team of authors.

## Results

### Quantitative Results

A total of 64 support workers and 35 young service users responded to the survey. The closed questions suggested that most responding young service users and support workers had access to digital devices (97–100%). Most of the support workers reported that they could get technical support from a central department or colleagues (78–83%), and some stated that they managed by themselves (56%). While many young service users reported that they managed without technical support (63%), several support workers (55%) indicated that they helped with some technical issues.

The most frequently reported forms of digital communication were telephone calls and text messages (54–69% for young service users and 100% for support workers). Other forms were less frequently reported by the young service users, while several support workers reported that they also used video calls (42%), camera functions (39%), shared calendars (22%), and chat functions (17%). Several of the young service users who reported that they had remote support stated that this practice was initiated by themselves or in a joint decision with the support worker. This was corroborated by the support workers, although some stated that the decisions in some cases were made on an organizational level (Online Resource, Table S3). Overall, the young service users’ needs for technical support, use of digital communication mode, and initiative for remote support were similar for the diagnostic groups.

The survey indicated that all included support areas were relevant, with “Solving problems/making decisions”, “Carrying out daily routines”, and “Doing housework” being the most frequently targeted areas, and the least frequently being “Economic self-sufficiency” and “Recreation and leisure”. Targeted support areas were broadly similar for the diagnostic groups, with slightly lower numbers for the autism group in the areas “Recreation and leisure” and “Energy and drive functions”. Remote support was frequently used in all areas according to both stakeholder groups. Whether or not those with remote support wanted to continue using that format differed, with support workers indicating a much greater interest (81–92%) than young service users (42–68%). Most support workers who provided support remotely stated that they wanted to continue doing so in the future. However, a few indicated that they had no interest in using remote support. Of those who did not provide support remotely, many expressed that they would like to do so (Online Resource, Tables S4-S5).

**Table 2:** Joint display of the integration of qualitative and quantitative findings with meta-inferences

Overarching themes	Qualitative inferences	Quantitative inferences	Meta-inferences
Person-centered service	• In-person meetings should not be replaced by remote support	• Remote support may be initiated by both service users and support workers	• Remote support can be an important complement to traditional housing support
	• Remote support may be a possible complement to regular support	• Some service users may need technical support to enable the use of remote support	• It is important to ensure that the social and emotional needs of service users are not neglected
	• Use of remote support may help empower some service users	• Remote support could be relevant for a range of service areas	• The service must respond to each service user’s preferred mode and style of communication
	• Digital technology may increase accessibility and user choice	• Support workers may be more interested than the service users in remote support	• For remote support to be accessible for all, help with technical issues must be available when needed
	• Social and emotional contact may be a key component of the support		• Engaging the service users in shared decision-making seems crucial in planning and implementing remote support elements
	• Remote communication can be complicated		
	• Remote support may be better suited for some individuals and specific support needs		
Equitable service	• Remote support should be functional and structured to ensure quality	• Access to digital devices and IT support seems to be good	• Coordinated efforts to manage organizational barriers will be crucial to ensure a quality of service that is adequate and equal for all
	• Increased knowledge and skills in digital technology and remote communication among support workers will be needed	• The initiative to use remote support may in some cases be taken on an organizational level	• To facilitate remote support, necessary infrastructure, and service routines need to be established
	• Organizational factors may constitute barriers to remote support	• Some support workers may prefer not to use remote support at all	• Educational needs among the staff must be met
	• Individual support needs may go unnoticed in remote contact		• It is important to ensure that staff attitudes do not lead to an unequal service provision
			• When using remote support, it is important to strive for clarity, balance, and timing

### Qualitative Results

A total of 55 support workers and 30 service users chose to provide free-text responses to one or more of the questions in the survey. This information was supplemented with in-depth information from the focus groups. The results are presented for the total sample of participating young adults, as the expressed views and experiences generally were similar across diagnostic groups. Four categories with a total of nine sub-categories were identified: Attitudes (Concerns. Complement, Empowerment); Accessibility (Possibilities, Barriers); Interpersonal interactions (Social and emotional contact, Remote communication), and Support needs (Clarifying needs, Activities of daily living). The comprising elements of each sub-category are presented below along with pertinent quotes from the survey marked with response number, stakeholder group, and diagnosis.

#### Attitudes

This category captures attitudes towards remote support, including concerns about replacing in-person meetings, seeing possibilities to complement current practices, and recognizing the potential to empower young adults.

*Concerns.* Overall, the service users expressed predominantly negative attitudes towards remote support, and some stated that they would rather cease the support service than only have remote contact. A few reported that they would accept remote support.

*“Remote support would ruin the whole point of a dedicated person dedicating their time.” 31 – Young adult, autism and ADHD*.

*“I think you should always try something new.” 1 – Young adult, ADHD*.

The support workers were generally more positive. Still, many support workers expressed a preference for in-person meetings, and that remote support does not suit all young adults or support workers.

*Complement.* Several support workers noted that the form of support should be requested by the young adults based on their preferences, needs, and current situation.

*“All should be at the service user’s request, of course.” 11 – Support worker*.

A few young adults expressed that different forms of support are needed. Both stakeholder groups emphasized the importance of tailoring the support.

*“Remote support is a good option. I think it needs to be combined with “regular” support in some cases.” 24 – Support worker*.

*Empowerment.* Both stakeholder groups suggested that remote support could be used to maintain skills and structures while gradually phasing out the support. Most support workers and a few service users saw remote support as a possibility to develop autonomy.

*“By offering remote support, I also believe that we can strengthen our clients to become a little more independent, i.e., not as dependent on us. Perhaps remote support can also be used as a form of “maintenance support” during a period when a person is about to end their support.” 9 – Support worker*.

#### Accessibility

This category covers the increased opportunities for contact and support when using digital technology, but also highlights technological, organizational, and individual difficulties.

*Possibilities.* Some support workers believed that digitally delivered support would be feasible when working with younger people. Both stakeholder groups saw the usefulness of digital devices to share information, tips, and explanations, as well as the advantages of being able to have digital contact when in-person meetings were challenging or not possible. Increased accessibility and flexibility (e.g., support outside regular support hours and effective rescheduling) were suggested.

*“Providers can work in new ways with more short visits via link.”43 – Support worker*.

According to both stakeholder groups, the provision of timely support for time-sensitive problems could sometimes be possible through digital contact, which could contribute to a sense of security.

*“If I get stressed when I have to perform an activity, I would like to be able to call (for example) to get help there and then.” 33 – Young adult, autism*.

Remote support was also described as time-efficient and may contribute to the continuity of the support.

*“The times I have offered support remotely, it has led to fewer cancellations.” 26 – Support worker*.

*Barriers.* The need for increased knowledge and skills in digital technology among the support workers was highlighted by both stakeholder groups.

*“The support workers’ skills must be improved to provide adequate remote support.” 7 – Support worker*.

According to the focus groups, various practical aspects may also determine whether remote support was provided (e.g., approved channels, adaptation of facilities to digital conversations, and resource allocation).

Other barriers experienced by service users were software problems, lack of network connection, and added costs. The support workers reported that they gave some technical guidance to enable remote support, e.g., support with hardware, apps, text messages, and digital meetings. In addition, both stakeholder groups mentioned that digital contact may be stressful and anxiety-provoking for some young adults, which potentially could constitute a barrier to support provision.

*“I wouldn’t accept support as often, because I am afraid of the phone.” 18 – Young adult, ADHD*.

Some additional aspects were raised by the focus groups. The support workers believed that there is a need for openness regarding difficulties using technology, and both stakeholder groups identified a need for explicit routines and a clear structure for remote support. For instance, the young adults pointed out that they did not want to bother their support worker and may refrain from initiating contact unless the routines for such contact were clear. The support workers raised that the expectations to always be available can be stressful and make their work more unpredictable. This may force the support workers to make spur-of-the-moment decisions about time allocation, which may lead to an unfair distribution of support.

#### Interpersonal Interaction

This category deals with the impact of remote support on the social and emotional contact and communication between the service user and the support worker.

*Social and emotional contact.* Both stakeholder groups expressed that in-person meetings were necessary to build trusting relationships and a good working alliance.

*“I believe that our work needs to consist of relationships based on trust. Which I still believe we can best achieve by meeting in person.” 24 – Support worker*.

There were concerns that digital contact may feel less personal and create a sense of distance. According to the focus group of support workers, meeting another person and having continuous contact is at the core of the support service, including building a relationship, adapting one’s behavior, and setting boundaries.

Both stakeholder groups pointed out that human contact has an energy or power that might be missing in digital service encounters. They emphasized the “emotional presence” of in-person meetings as a factor that may help reduce young adults’ stress and provide a sense of security.

*“I prefer in-person meetings. It is more difficult to support me over the phone with my anxiety about, for example, going shopping or taking out the garbage.” 2 – Young adult, autism*.

The need for in-person meetings to promote social connection and reduce social isolation was described by both stakeholder groups.

*“I tend to isolate myself because I have social anxiety. So, I need meetings on-site to get out more.” 20 – Young adult, autism and ADHD*.

On the other hand, both stakeholder groups expressed that hosting someone in their home takes energy, and that remote contact may reduce the effort.

*“Some of my service users have clearly expressed that it is easier to do things if we talk on the phone instead of me being on-site because it can feel overwhelming to have someone watching you when you do the dishes, for instance.” 11 – Support worker*.

*Remote communication.* Communicating remotely was described by both stakeholder groups as a complex task. A few described communications to be easier through digital channels while many pointed out that non-verbal cues were less clear in remote communication.

*“Communication is not the same over the phone as IRL.” 6 – Young adult, autism*.

The focus group of young adults described that the ‘tone’ could be perceived as harsh or difficult to interpret over the phone, particularly when facial expressions and body language were absent. They also pointed out that joking on the phone should be avoided, as it may lead to misunderstandings. The importance of quiet moments during a conversation was also highlighted, something that was perceived as more difficult over the phone. Additionally, they mentioned that support workers should be fully present in the conversation and avoid multitasking (e.g., driving a car while providing support by phone). Overall, both stakeholder groups believed that delivering support remotely placed greater demands on the support worker’s communication skills. Written communication (e.g., text messages or e-mails) was described as more time-efficient and less personal, which was noted as a positive aspect across all three focus groups. On the other hand, service users highlighted that at times they struggled to interpret messages (e.g., smileys) and that formulating a text could be both time- and energy-consuming.

#### Support Needs

This category captures difficulties in clarifying needs when communicating remotely and that the level of functionality should be factored in when deciding whether to use remote elements or not.

*Clarifying needs.* Both stakeholder groups believed that using remote support required transparency about current well-being and challenges in daily living to avoid important support needs going unnoticed. When this is not possible, in-person support may help the support worker observe subtle details about the individual and the environment.

*“It’s easy to lie and say you’re fine when in fact you look like a mess and don’t have the energy to make contact.” 6 – Young adult, autism*.

*“It is easier to feel the atmosphere during on-site meetings, easier to read body language, to see the environment, what things are and aren’t there, to help pay attention to different things in the room, to see a need for support that is not visible digitally.” 29 – Support worker*.

*Activities of daily living.* Many service users described that remote support challenged their cognitive functioning, where they risked not getting anything done because they forgot, had difficulties getting started, or were unable to implement new strategies. They described that it was more difficult to stay focused, solve problems, and remain engaged when nobody was there to keep them focused on the activity. However, some pointed out that support on-site also could be a barrier if the support worker was interrupting their focus on an activity.

*“I don’t do what I should if I only get reminders by phone. I might as well use the reminder settings on my phone. If that had worked, I wouldn’t have needed support in daily living.” 5 – Young adult, ADHD*.

According to the focus groups of young adults, the very presence of another person could make a significant difference to a support meeting making it clearer and ‘more real’ and providing a ‘positive influence’ that could promote action. They talked about a ‘body doubling’ phenomenon, which was also reflected in the young adults’ survey responses.

*“If I don’t have my support worker in the room, I don’t manage to do what I want to get done.” 5 – Young adult, ADHD*.

This was echoed by the support workers, suggesting that on-site support had advantages when initiating an activity or giving instructions. According to the focus group of support workers, managing sensory sensitivities (e.g., contact with water or dirt) could be difficult to do remotely.

Both stakeholder groups suggested that remotely delivered support elements (e.g., behavior activation, planning, reminders, follow-ups of activities, motivational talks, and digital presence) may be effective complements in some support areas (e.g., daily routines, health, finances, leisure time, and use of time and energy).

*“I can often complete daily tasks with remote support.” 33 – Young adult, autism*.

Some more practical tasks were described as better suited for on-site support (e.g., sensitive financial issues and important meetings).

*“Support is often needed in interactions with different authorities or healthcare providers and our presence is needed as many of our service users are met with distrust.” 28 – Support worker*.

The support workers also highlighted that many young adults may need support on-site in using digital technology in their daily lives (e.g., using authorities’ e-services, navigating websites, and using button selection).

### Integration of Quantitative and Qualitative Findings

Two recurrent themes aligned with the WHO framework (World Health Organization, 2018) were identified across the qualitative and quantitative findings: *Person-centered service* and *Equitable service*. Meta-inferences are presented in the joint display (Table 2).

## Discussion

This is the first study to investigate how neurodivergent young service users and support workers view the use of remote elements in support in daily living. We found that while access to digital devices was reported to be good, adequate service routines for remote contact were not yet in place. Young service users were more hesitant than the support workers in endorsing the shift towards remote contacts, expressing concerns that remote contact would lead to substandard support. Still, both support workers and young service users felt that remote contact could be a valuable complement to in-person meetings, increasing accessibility and user choice. Overall, our findings closely align with what previously has been found in health care settings (World Health Organization, 2019).

The two recurring themes from the integration of qualitative and quantitative findings (i.e., *person-centered service* and *equitable service*) underscore the imperative to carefully consider the quality of service when implementing remote support. These two themes are closely related to two key aspects of the World Health Organization’s framework for quality elements in health services: people-centeredness (i.e., responding to a person’s preferences, needs, and values, and involving the person in the development of their implementation plan) and equity (i.e., reflect evidence on the potential benefits of the intervention only, and nothing else) (World Health Organization, 2018).

Communication and human interaction stood out as crucial factors in ensuring a person-centered service. Communication is a complex process, where the ability to convey and receive verbal and non-verbal information are key components. Both stakeholder groups expressed that the quality of remote communication partly depended on the support workers’ communication skills. This has previously been discussed by Tang et al. (2021), regarding remotely delivered motivational interviewing. The importance of specific skills in remote communication was highlighted, like managing the absence of non-verbal cues and being comfortable sitting in silence. An understanding of each young service user’s preferences is also required. For instance, some neurodivergent individuals may experience written text as less demanding than real-time communication (e.g., Ali et al., 2023; Cummins et al., 2020; Sehlin et al., 2018). On the other hand, perception of written text (e.g., understanding norms and jokes) (Cummins et al., 2020) or the use of emojis and other symbols (Hand et al., 2023), can be demanding and lead to uncertainty. In addition, body language, details in the home, or other non-verbal clues can convey important information about the young service users’ current well-being and overall situation (Ali et al., 2023; Zaagsma et al., 2022). Such signals may be lost in remote communication. Overall, this emphasizes that there are many aspects to consider in achieving well-functioning remote communication.

Similarly, both stakeholder groups stressed the importance of human interaction. This is in line with previous findings suggesting that the relationship and social interaction between service users and their support workers is a crucial dimension of support in daily living, which goes beyond the practical tasks (Andersson & Gustafsson, 2019). This also relates to the broader discussion on digital health care where the lack of social interactions has been highlighted as a drawback (Beland et al., 2022; Fang et al., 2022; Stevens et al., 2019). In the present study, concerns were raised that remote contact can result in more practically oriented support with fewer opportunities for social facilitation. Possible benefits of blended remote and in-person support were expressed by both stakeholder groups, something that has previously been recognized as a preferable approach in health care and community services (e.g., Chen et al., 2022; Fang et al., 2022; Khan et al., 2019; Venturo-Conerly et al., 2022). Individual differences in human interaction needs underscore the importance of engaging service users in planning support. This could ensure that significant aspects of genuine human contact are not missed, such as engagement, being understood, and feeling a sense of belonging (e.g., Bright & Doody, 2023; Krane et al., 2023).

While the accessibility of digital devices provides new opportunities for contact and access to support, it may also lead to inequities in service provision. Varying competencies and unclear infrastructure were two key aspects raised by the stakeholders that may affect service equality. The necessity of technical literacy among service providers has become topical after the COVID-19 pandemic (e.g., Ali et al., 2023; Fang et al., 2022). To ensure the quality of support services, both stakeholder groups requested improved skills among support workers in using digital technology and remote communication. Insufficient staff training in the use of digital technology has previously been pinpointed as a barrier when giving support to neurodivergent adults (Khanlou et al., 2020). Regardless of technical skills, though, not all the support workers seemed to embrace digital technology in the provision of support.

While remote contact may increase flexibility and efficiency (e.g., more frequent and timely contacts), challenges were also raised related to data protection and unclear expectations and boundaries around accessibility (e.g., being available all the time). Both stakeholder groups highlighted a need for transparency about such factors to bring clarity, balance, and timing to the implementation of remote support elements.

Finally, while today’s neurodivergent young service users may be used to digital technology and remote contact, they may not necessarily embrace remote services (Adamou et al., 2021). In addition, telephone anxiety has been reported to be a major concern among young adults (Howard & Sedgewick, 2021; Kim & Oh, 2023). Therefore, further exploration of young service users’ preferences and needs will be necessary when planning and implementing personalized support services, including a more comprehensive mapping of demand and acceptability. In addition, it is important to keep in mind that current perspectives may change over time and may be impacted by future technological advances. To promote the development and implementation of sustainable services, where service users can choose and control their support, person-centeredness must be addressed at both the individual and organizational levels (Nolte et al., 2020). In developing such a service model, the involvement of several stakeholder groups (e.g., young adults, support workers, and managers) will be required.

### Limitations

While this study expands our understanding of how remote support in daily living is perceived, the results should be interpreted with caution due to some limitations. Firstly, the included sample may differ from the target populations of young service users and support workers in significant ways. It is for instance possible that the decision to participate in the survey was partly influenced by participants’ pre-existing attitudes. In addition, while both online and paper versions of the survey were available for the service users, some may prefer non-text communication to share their views. For instance, the views of the subgroup of service users and support workers declining to provide free-text responses may not be captured in our findings. Judgement about generalizability/transferability of the survey findings is further complicated by the fact that no information was available on some relevant characteristics (e.g., educational level and co-occurring conditions). This was true not only for our sample but also for the broader target population. In addition, the exact number of service users and support workers approached for the survey was not available. Judging by the relatively large proportion of female respondents, it is unlikely that the sample was representative of all young neurodivergent service users. Although a wide range of educational backgrounds and occupational statuses were represented in the focus groups, the views expressed may not fully reflect those of the target population. At a minimum, though, our results show that subgroups of young service users express concerns about the current development.

Secondly, the number of participants was relatively small, especially when categorized into diagnostic subgroups. In addition, these conditions frequently co-occur (Antshel & Russo, 2019). While no clear contrast was observed between autistic young adults and those with ADHD, we cannot rule out that there may still be important differences. The same is true for other characteristics, such as age, gender, educational background, and co-occurring conditions. Larger samples and strategies to ensure a more representative group of respondents, including a balanced gender ratio, will be needed to further explore individual differences. Future research should also ensure that neurodivergent service users are involved throughout the whole research process, to enhance inclusivity, improve the quality and relevance of the findings, and capture a wider range of perspectives (Fletcher-Watson et al., 2019; National Institute for Health and Care Research, 2021).

Thirdly, although the research team’s familiarity with the topic may have been an advantage in the analysis (Krippendorff, 2019), there is also a risk that our pre-understanding may have led us to overlook some relevant aspects. In addressing this, the authorship team’s varying levels of knowledge and experience of the support services were utilized to facilitate a nuanced interpretation. In addition, our use of multiple data collection methods with varying degrees of researcher involvement may have reduced researcher bias (Creswell, 2022).

## Conclusion

Technological advances may inevitably lead to significant changes in how support in daily living will be structured. Service providers planning a shift to increased use of remote support in daily living should be aware that not all young service users embrace this new service model. Providers must also consider organizational readiness, including the need for guidance, education, and improved infrastructure. With a more complex and diverse support service, user engagement and shared decision-making will be crucial to meet individual preferences, values, and needs.

## Electronic supplementary material

Below is the link to the electronic supplementary material.

Supplementary Material 1
