# Supplementary material for: ‘If I Don’t Have My Support Worker in the Room…’: A Multi-perspective Mixed Methods Study of Remote Daily Living Support for Neurodivergent Young Adults
Source: J Autism Dev Disord. 2024 Jun 5;55(9):3140–53. doi: 10.1007/s10803-024-06425-z (PMC12367811; doi:10.1007/s10803-024-06425-z)
Supplement: Supplementary file 1 — Supplementary Material 1 [file 10803_2024_6425_MOESM1_ESM.pdf]

Online Resource: Supplemental tables

'If I don't have my support worker in the room…': A multi-perspective mixed methods study of remote daily living support for neurodivergent young adults

Content

Table S1. Characteristics for young service users responding to the survey, stratified by diagnostic subgroup. 2

Table S2. Example of branching question for young service users and support workers. The italicized text shows web survey construction. 3

Table S3. Young service users' and support workers' use of digital devices in housing support. 4

Table S4. Support in various support domains, current use of remote support, and future interest in remote support. 5

Table S5. Young service users' support in different support areas, current use of remote support and future interest in remote support stratified by diagnostic subgroup. 6

Table S1. Characteristics for young service users responding to the survey, stratified by diagnostic subgroup.

		Total	Autism	ADHD	Autism and ADHD
	All[a]	35	16	10	8
Gender	Women	21	10	5	5
	Men	10	5	2	3
	Non-binary	4	1	3	0
Age[b]	18–24 years	9	5	2	2
	25–29 years	25	11	8	5
Time with support in daily living	≈ 1 year	12	8	3	1
	≈ 2–3 years	17	5	7	5
	≈ 4–10 years	6	3	0	2

[a]One respondent did not state their diagnosis.
[b]One respondent did not state their age.

Table S2. Example of branching question for young service users and support workers. The italicized text shows web survey construction.

Question for young service user	Question for support worker
Do you get support in:<br>9. Are you supported in using your energy for what you want and need to do?	Do you support people with ADHD/autism in:<br>9. ... using their energy for what they want and need to do?
Tick Yes / No	*Tick* Yes / No
9.1. and 9.2. opens in case the response on 9 is Yes<br>9.1. If Yes - are you supported remotely with this?<br>*Tick* Yes / Partially / No	*9.1. and 9.2. opens in case the response on 9 is Yes*<br>9.1. If Yes – do you provide this support remotely?<br>*Tick* Yes / Partially / No
9.2. Regardless of your support today, do you want to get remote support in the future?<br>*Tick* Yes / Partially / No<br><br>Please describe why in your own words:<br>*Free text*	9.2. Regardless of how you provide support today - do you want to provide this support remotely in the future?<br>*Tick* Yes / Partially / No<br><br>Please describe why in your own words:<br>*Free text*

Table S3. Young service users' and support workers' use of digital devices in housing support.

	**Young service users,** number (%)	**Support workers,** number (%)
In total	35	64
Access to digital devices (n)		
Telephone (incl smartphone)	34[a] (97)	64 (100)
Computer (desktop or laptop)	34[a] (97)	64 (100)
Tablet	11[a] (31)	23 (36)
Access to IT support[a] (support workers only)		
Central IT department	N/a	53 (83)
Colleagues	N/a	50 (78)
Others	N/a	8 (13)
Manage by myself	N/a	36 (56)
Service users' need for technical support[a] / Support workers providing technical support to young service users		
No support	22 (63)	27 (42)
Some support	5 (14)	35 (55)
Much support	0 (0)	2 (3)
Do not know	7 (20)	N/a
Digital forms used in the support		
Telephone calls	19 (54)	64 (100)
Text messages (SMS and MMS)	24 (69)	64 (100)
Video calls	1 (3)	27 (42)
Shared calendar	1 (3)	14 (22)
Chat functions	0 (0)	11 (17)
Camera functions	1 (3)	25 (39)
On whose initiative is remote support used[b]		
Service user's suggestion	6 (17)	32[c] (50)
Support worker's suggestion	7 (20)	20 (31)
Joint suggestion	8 (23)	N/a
Support form not an option for me	12 (34)	N/a
Colleague's suggestion	N/a	2 (3)
Service provider´s decision	N/a	9 (14)

[a] Missing data for one respondent
[b] Missing data for two service users and one support worker
[c] On whose initiative it usually is (given that support workers provide support to several service users)

Table S4. Support in various support domains, current use of remote support, and future interest in remote support.

ICF code	Young service users (n=35)			Support workers (n=64)		
	**Currently supported, number (%)**	**Remote support[a], number (%)**	**Future interest in remote support[a], number (%)**	**Currently providing support, number (%)**	**Remote support[a], number (%)**	**Future interest in remote support[a], number (%)**
Solving problems/ making decisions	33 (94)	21 (64)	20[b] (61)	64 (100)	47[b] (75)	55 (86)
Carry out daily routines	28 (80)	14[b] (50)	18[b] (64)	62 (97)	44[b] (70)	57 (92)
Handling stress, psychological demands	24[b] (68)	11 (46)	14 (58)	52[c] (81)	37[b] (73)	45 (86)
Looking after one's health	18[b] (53)	9 (50)	12 (68)	57 (89)	39 (68)	50 (88)
Doing housework	31[b] (88)	10 (32)	13[b] (42)	60 (94)	39 (65)	52 (87)
Basic interpersonal interactions	17[b] (49)	8 (47)	8 (47)	49 (77)	23[b] (48)	41 (84)
Economic self-sufficiency	12[b] (34)	6 (50)	6 (50)	49 (77)	26 (53)	43 (88)
Recreation and leisure	12[b] (34)	6 (50)	8 (67)	47 (73)	22[b] (48)	38[b] (81)
Energy and drive functions	22[b] (63)	11 (50)	12 (54)	58 (91)	36 (62)	48[b] (83)
People in positions of authority	28[b] (80)	13[b] (46)	14 (50)	54 (84)	36 (67)	48 (89)

[a]Out of those currently supported/providing support in the domain.
[b]Missing data for one respondent.
[c]Missing data for three respondents.

Table S5. Young service users' support in different support areas, current use of remote support and future interest in remote support stratified by diagnostic subgroup.

	**Autism (n=16)**			**ADHD (n=10)**			**Autism and ADHD (n=8)**		
**ICF code**	**Currently supported, number (%)**	**Remote support[a], number (%)**	**Future interest in remote support[a], number (%)**	**Currently supported, number (%)**	**Remote support[a], number (%)**	**Future interest in remote support[a], number (%)**	**Currently supported, number (%)**	**Remote support[a], number (%)**	**Future interest in remote support[a], number (%)**
Solving problems/ making decisions	15 (94)	7 (47)	9[b] (60)	9 (90)	6 (67)	3 (33)	8 (100)	7 (88)	7 (88)
Carry out daily routines	11 (69)	5[b] (45)	7 (64)	9 (90)	3 (33)	3 (33)	7 (88)	5 (71)	7 (100)
Handling stress, psychological demands	9[b] (56)	3 (33)	5 (55)	6 (60)	2 (33)	3 (50)	8 (100)	5 (62)	5 (62)
Looking after one's health	7 (44)	2 (29)	4 (57)	6 (60)	3 (50)	4 (67)	4 (50)	3 (75)	3 (75)
Doing housework	12 (75)	2 (17)	6[b] (50)	10 (100)	2 (20)	1 (10)	8 (100)	5 (62)	5 (62)
Basic interpersonal interactions	5 (31)	0 (0)	1 (20)	4 (40)	1 (25)	0 (0)	7 (88)	6 (86)	6 (86)
Economic self-sufficiency	4 (25)	1 (25)	1 (25)	3 (30)	0 (0)	0 (0)	4 (50)	4 (100)	4 (100)
Recreation and leisure	1 (6)	0 (0)	0 (0)	5 (50)	1 (20)	3 (60)	5 (63)	4 (80)	4 (80)
Energy and drive functions	6 (38)	3 (50)	4 (67)	8 (80)	3 (38)	3 (38)	7 (88)	4 (57)	4 (57)
People in positions of authority	10 (62)	2 (20)	4 (40)	9 (90)	4 (44)	3 (33)	8 (100)	6 (75)	6 (75)

[a]Out of those currently supported/providing support in the domain.
[b]Missing data for one respondent
